# Report from the 5th cardiovascular outcome trial (CVOT) summit

**DOI:** 10.1186/s12933-020-01022-7

**Published:** 2020-04-17

**Authors:** Oliver Schnell, Eberhard Standl, Xavier Cos, Hiddo JL Heerspink, Baruch Itzhak, Nebojsa Lalic, Michael Nauck, Antonio Ceriello

**Affiliations:** 1 Forschergruppe Diabetese e. V., Ingolstaedter Landstraße 1, Neuherberg, 85764 Munich, Germany; 2 Sant Marti de Provençals Primary Care Centres, Barcelona, Spain; 3 Department of Clinical Pharmacy and Pharmacology, University Medical Center Groningen, University of Groningen, Groningen, The Netherlands; 4 George Institute for Global Health, George Institute, Camperdown, Sydney, NSW Australia; 5 Clalit Health Services and Technion Faculty of Medicine, 3 Zivoni Street, Haifa, Israel; 6 Faculty of Medicine, University of Belgrade, Clinic for Endocrinology, Diabetes and Metabolic Diseases, Clinical Center of Serbia, Dr Subotica 13, Belgrade, 11000 Serbia; 7 Diabetes Division, St. Josef-Hospital (Ruhr University), Bochum, Germany; 8 Department of Cardiovascular and Metabolic Diseases, IRCCS MultiMedica, Via Milanese 300, 20099 Sesto San Giovanni (MI), Italy

**Keywords:** Cardiovascular risk, Diabetes, CVOT, CAROLINA, CREDENCE, DAPA-HF, REWIND, PIONEER-6, SGLT-2 inhibitor, GLP-1 receptor agonist

## Abstract

The 5th Cardiovascular Outcome Trial (CVOT) Summit was held in Munich on October 24th–25th, 2019. As in previous years, this summit served as a reference meeting for in-depth discussions on the topic of recently completed and presented CVOTs. This year, focus was placed on the CVOTs CAROLINA, CREDENCE, DAPA-HF, REWIND, and PIONEER-6. Trial implications for diabetes management and the impact on new treatment algorithms were highlighted for diabetologists, cardiologists, endocrinologists, nephrologists, and general practitioners. Discussions evolved from CVOTs to additional therapy options for heart failure (ARNI), knowledge gained for the treatment and prevention of heart failure and diabetic kidney disease in populations with and without diabetes, particularly using SGLT-2 inhibitors and GLP-1 receptor agonists. Furthermore, the ever increasing impact of CVOTs and substances tested for primary prevention and primary care was discussed. The 6th Cardiovascular Outcome Trial Summit will be held in Munich on October 29th–30th, 2020 (https://www.cvot.org).

## Background

Diabetes mellitus (DM) presents an ever increasing burden of our time. Within the next 25 years, the International Diabetes Federation (IDF) estimates an escalation of patient numbers starting at a 15% increase of persons with DM in Europe, over a 33% increase in North America and the Caribbean to a 74%, 96%, and even a 143% increase in South-East Asia, the Middle-East and North Africa, and Africa, respectively [1]. Cardiovascular disease (CVD) such as, but not limited to, stroke, myocardial infarction (MI), atherosclerosis, heart failure (HF), coronary heart disease (CHD), angina pectoris, and cardiovascular (CV) death present major comorbidities of DM. A recent systemic literature analysis with evidence on over 4.5 million persons with type 2 diabetes mellitus (T2DM) across the globe revealed a prevalence of ≈ 32% CVD, ≈ 29% atherosclerosis, ≈ 21% CHD, ≈ 15 HF, ≈ 10% MI, and ≈ 7.5% stroke [2]. Consequently, CVD-related deaths represented 50.3% of all T2DM-related deaths [2]. Similarly, it has been proposed that at least 50% of all persons with T2DM worldwide have diabetic kidney disease (DKD) [3]. It has been shown that patients with chronic kidney disease (CKD) have an ≈ 18–47% increased mortality, depending on development of albuminuria and/or decline of glomerular filtration rate (GFR) [4]. In summary, this mandates affordable, accessible, but most importantly effective and save means of glycaemic control.

As some glucose-lowering medications raised concerns of elevated micro- and macrovascular risk, the American Food and Drug Administration (FDA) mandated Cardiovascular Outcome Trials (CVOTs) in diabetes in 2008, to prevent an undesired increase of CV risk [5]. Thus, approved glucose-lowering substances have undergone a CVOT to analyse pre-specified CV endpoints since, usually investigating a combined primary endpoint of CV death, non-fatal stroke, non-fatal MI (3-point major adverse cardiovascular event; 3P-MACE) and several pre-specified CV and/or renal secondary endpoints. So far, most CVOTs in diabetes were conducted for 3 substance classes emerging in the last 2 decades: dipeptidyl peptidase 4 inhibitors (DPP-4is; alogliptin [6], linagliptin [7], saxagliptin [8], and sitagliptin [9]), sodium/glucose co-transporter 2 inhibitors (SGLT-2is; canagliflozin [10], dapagliflozin [11], empagliflozin [12]), and glucagon-like 1 receptor agonists (GLP-1 RAs; albiglutide [13], exenatide [14], liraglutide [15], lixisenatide [16], and semaglutide [17]).

In 2019, the list of CVOTs in diabetes was expanded by 3 CVOTs (CAROLINA [18]—linagliptin; REWIND [19]—dulaglutide; PIONEER-6 [20]—oral semaglutide), a renal outcome trial (CREDENCE [21]—canagliflozin), and a HF outcome trial in patients with HF and reduced ejection fraction (HFrEF) with and without diagnosed DM (DAPA-HF [22]—dapagliflozin). Also, a renal trial on an endothelin A receptor antagonist (SONAR [23]—atrasentan) was published. In addition, a trial on angiotensin-neprilysin inhibition in HF with preserved ejection fraction (HFpEF; PARAGON-HF [24]—sacubitril-valsartan) was published. As in previous years [25–28], we present and summarise key aspects discussed at the 5th CVOT Summit in October 2019. The 5th CVOT Summit was an interdisciplinary platform and was held in conjunction with four study groups of the European Association for the Study of Diabetes (EASD): the Diabetes and Cardiovascular Disease EASD Study Group (DCVD, www.dcvd.org), Primary Care Diabetes Europe (PCDE, www.pcdeurope.org), European Diabetic Nephropathy Study Group (EDNSG, www.ednsg.org), and the Incretin study group. Participants from 4 continents with specialities in endocrinology & diabetology, cardiology, nephrology, and primary care contributed to the discussions of the 5th CVOT Summit in 2019 (www.cvot.org).

### Updates on CVOTs

A summary of characteristics and results of renal, HF and CV outcome trials published in 2019 is listed in Tables 1, 2, 3, and 4.

**Table 1:** Overview of basic characteristics of renal, heart failure and cardiovascular outcome studies completed in 2019

Study name	Study status	Drug	Drug class	Intervention	Primary outcome	N	Follow up [years]	Start and end date	Clinicaltrials.gov ID
CAROLINA [18]	Completed	Linagliptin	DPP-4 inhibitor	Linagliptin 5 mg once daily vs. Glimepiride 1-4 mg	CV-death, non-fatal MI, non-fatal stroke	6.042	6.3	11.2010–08.2018	NCT01243424
PIONEER-6 [20]	Completed	Semaglutide oral	GLP-1 receptor agonist	Semaglutide oral 14 mg once daily vs. placebo	Death from CV causes (including undetermined causes of death), non-fatal MI, non-fatal stroke	3.183	1.3	01.2017–09.2018	NCT02692716
REWIND [19]	Completed	Dulaglutide	GLP-1 receptor agonist	Dulaglutide 1.5 mg weekly vs. placebo	Non-fatal MI, non-fatal stroke, death from CV causes or unknown causes	9.901	5.4	07.2011–08.2018	NCT01394952
CREDENCE [21]	Completed	Canagliflozin	SGLT-2 inhibitor	Canagliflozin 100 mg once daily vs. placebo	End-stage kidney disease, sustained doubling of serum creatinine level, death from renal or CV disease	4.401	2.6	02.2014–10.2018	NCT02065791
DAPA-HF [22]	Completed	Dapagliflozin	SGLT-2 inhibitor	Dapagliflozin 10 mg once daily vs. placebo	Worsening heart failure or death from CV causes	4.744	1.5	02.2017–07.2019	NCT03036124

**Table 2:** CVOTs completed in 2019: comparison of results

Cardiovascular endpoints	CAROLINA [18]		PIONEER-6 [20]		REWIND [19]	
	Class	HR (95% CI) p-value	Class	HR (95% CI) p-value	Class	HR (95% CI) p-value
Primary composite outcome	CV-death, non-fatal MI, non-fatal stroke	0.98 (0.84–1.14) p < 0.001 (non-inferiority) p = 0.76 (superiority)	Death from CV causes (including undetermined causes of death), non-fatal MI, non-fatal stroke	0.79 (0.57–1.11) p < 0.001 (non-inferiority) p = 0.17 (superiority)	Death from CV causes or unknown causes, non-fatal MI, non-fatal stroke	0.88 (0.79–0.99)^a^ p = 0.026 (superiority)
Cardiovascular death	Secondary or tertiary outcome	1.00 (0.81–1.24)	Secondary outcome^b^	0.51 (0.31–0.84)^b^	Secondary outcome	0.91 (0.78–1.06) p = 0.21
Myocardial infarction (fatal and non-fatal)	Secondary or tertiary outcome	1.03 (0.82–1.29)	Non-fatal MI Secondary outcome	1.18 (0.73–1.90)	Secondary outcome	0.96 (0.79–1.15) p = 0.63
Stroke (fatal and non-fatal)	Secondary or tertiary outcome	0.86 (0.66–1.12)	Non-fatal stroke Secondary outcome	0.74 (0.35–1.57)	Secondary outcome	0.76 (0.62–0.94) p = 0.010
Hospitalisation for unstable angina	Secondary or tertiary outcome	1.07 (0.74–1.54)	Secondary outcome	1.56 (0.60–4.01)	Secondary outcome	1.14 (0.84–1.54) p = 0.41
Hospitalisation for heart failure	Secondary or tertiary outcome	1.21 (0.92–1.59)	Secondary outcome	0.86 (0.48–1.55)	Secondary outcome	0.93 (0.77–1.12) p = 0.46
All-cause death	Secondary or tertiary outcome	0.91 (0.78–1.06)	Secondary outcome	0.51 (0.31–0-84)	Secondary outcome	0.90 (0.80–1.01) p = 0.067
Other outcomes	4P-MACE^d^ Key secondary endpoint	0.99 (0.86–1.14)	Expanded composite outcome^c^	0.82 (0.61–1.10)	Composite microvascular outcome	Eye: 1.24 (0.92–1.68) p = 0.16
						Renal: 0.85 (0.77–0.93) p = 0.0004

	Event rate (%) linagliptin vs. glimepiride group		Event rate (%) active vs. placebo group		Event rate (%) active vs. placebo group	
Primary composite outcome	11.8 vs. 12.0		3.8 vs. 4.8		12.0 vs. 13.4	
*Adverse events*	*No. (%) linagliptin vs. glimepiride group* *p-value*		*No. (%) active vs. placebo group* *p-value*		*No. (%) active vs. placebo group* *p-value*	
Renal event	– –		2.0 vs. 2.3 –		1.7 vs. 1.9 0.46	
Acute pancreatitis	0.5 vs. 0.5 –		0.1 vs. 0.2 –		0.5 vs. 0.3 0.11	
Severe hypoglycaemic events	0.3 vs. 2.2^e^ –		1.4 vs. 0.8 –		1.3 vs. 1.5 0.38	

^a^After accounting for α = 0.009 spent on the primary outcome for the interim analysis, the α for the final analysis is 0.0467 [HR 0.88 (95.33% CI 0.79–0.99)]

^b^Death from cardiovascular causes

^c^Death from cardiovascular causes, non-fatal myocardial infarction, non-fatal stroke, unstable angina resulting in hospitalization, or heart failure resulting in hospitalization

^d^CV death, non-fatal myocardial infarction, non-fatal stroke, or hospitalization for unstable angina pectoris

^e^Requiring the assistance of another person to actively administer carbohydrate, glucagon, or other resuscitative actions

**Table 3:** Renal outcome trials completed in 2019: comparison of results vs. placebo

CREDENCE [21]			
Class and cardiovascular/renal endpoints	HR (95% CI) p-value	Event	Event rate (%) active vs. placebo group
Primary composite endpoint		Primary composite outcome	11.1 vs. 15.5
End-stage kidney disease, sustained doubling of serum creatinine level, death from renal or CV disease	0.70 (0.59–0.82) p = 0.00001		
Secondary outcome		*Adverse events*	Event rate (%) active vs. placebo group (p-value)
CV death or hospitalization for heart failure	0.69 (0.57–0.83) p < 0.001	Renal event	3.9 vs. 4.5^a^ (–)
Secondary outcome		Acute pancreatitis	0.2 vs. < 0.1 (–)
CV death, myocardial infarction, or stroke	0.80 (0.67–0.95) p = 0.01	Diabetic ketoacidosis	0.5 vs. < 0.1 (–)
Secondary outcome			
Hospitalization for heart failure	0.61 (0.47–0.80) p < 0.001		
Secondary outcome			
End-stage kidney disease, doubling of serum creatinine level, or renal death	0.66 (0.53–0.81) p < 0.001		
Secondary outcome			
All-cause death	0.83 (0.68–1.02)		
Secondary outcome			
CV death, myocardial infarction, stroke, or hospitalization for heart failure or for unstable angina	0.74 (0.63–0.86)		
Secondary outcome			
Dialysis, kidney transplantation, or renal death	0.72 (0.54–0.97)		
Exploratory outcome			
Cardiovascular death	0.78 (0.61–1.00) p = 0.05		
Exploratory outcome			
Renal death	0.78 (0.61–1.00)		

^a^Acute kidney injury

**Table 4:** Heart failure outcome trials completed in 2019: comparison of results vs. placebo

DAPA-HF [22]			
Class and cardiovascular/renal endpoints	HR (95% CI) p-value	Event	Event rate (%) active vs. placebo group
Primary composite endpoint		Primary composite outcome	16.3 vs. 21.2
Worsening heart failure or death from CV causes	0.74 (0.65–0.85) p < 0.01		
Secondary outcome		*Adverse events*	No. (%) active vs. placebo group (p-value)
CV death or heart-failure hospitalization	0.75 (0.65–0.85) p < 0.001	Renal event	6.5 vs.7.2 (0.36)
Secondary outcome		Acute pancreatitis	– (–)
Total no. of hospitalizations for heart failure and CV deaths	0.75 (0.65–0.88) p < 0.001	Diabetic ketoacidosis	0.1 vs. 0^a^ (–)
Secondary outcome			
Change in KCCQ total symptom score at 8 months	1.18 (1.11–1.26) p < 0.001		
Secondary outcome			
Worsening renal function	0.71 (0.44–1.16)		
Secondary outcome			
All-cause death	0.83 (0.71–0.97)		
Exploratory outcome			
Cardiovascular death	0.82 (0.69–0.98)		

^a^All cases of diabetic ketoacidosis occurred in patients with diabetes at baseline

### DDP-4 inhibitors

The previously published CARMELINA trial [7] investigated CV effects of linagliptin compared to placebo (primary endpoint 3P-MACE, HR 1.02 [95% CI 0.89–1.17]) in patients with T2DM at high risk of CV and kidney events. The recently published CAROLINA trial [18] assessed CV outcomes of linagliptin compared to a sulfonylurea (glimepiride). Included patients were adults with T2DM, HbA1c of 6.5–8.5%, and high CV risk (1. established atherosclerotic CVD (ASCVD), 2.  ≥ 2 risk factors, 3. age ≥ 70 years, 4. evidence of microvascular complications). HbA1c of eligible participants currently receiving sulfonylurea or glinide as monotherapy or in combination with metformin or α-glucosidase inhibitors was restricted from 6.5 to 7.5% and sulfonylurea/glinide therapy was discontinued at randomization. The primary outcome of the CAROLINA trial was 3P-MACE, with an additional key secondary CV endpoint (4P-MACE) and two key secondary glycaemic endpoints (1. treatment and maintenance of HbA1c ≤ 7.0% at final visit without the need for rescue medication, without any moderate/severe hypoglycaemic episodes, and without > 2% weight gain; 2. treatment and maintenance of HbA1c ≤ 7.0% at final visit without the need for rescue medication and without > 2% weight gain) [18].

The primary endpoint was not significantly changed (HR 0.98 [95% CI 0.84–1.14]; p < 0.001 for non-inferiority; p = 0.76 for superiority), however, linagliptin demonstrated non-inferiority compared to glimepiride. As superiority was not observed, key secondary and other secondary/tertiary endpoints were presented descriptively only. Overall, linagliptin demonstrated CV safety, yet no CV benefit compared to glimepiride, also reflected in the endpoint of any adjudicated-confirmed CV event (HR 0.96 [95% CI 0.85–1.09]) [18]. However, on the other hand and equally important, it has been proposed subsequently that this trial not only demonstrated CV safety of linagliptin, but also provided reliable evidence for the CV safety of glimepiride [18, 29].

### SGLT-2 inhibitors

The CREDENCE trial [21] assessed the renal and CV outcomes in 4401 patients with T2DM and albuminuric CKD over a median follow-up of 2.62 years. Eligible patients were ≥ 30 years, had T2DM with a HbA1c of 6.5–12%, and CKD defined as eGFR ≥ 30 to < 90 ml/min/1.73 m^2^, and an urine albumin-creatinine ratio (UACR) of > 300 to < 5000 mg/g. In addition, all patients were required to be on a stable dose of angiotensin-converting-enzyme (ACE) inhibitor 1 month prior to randomization (dual-agent treatment with ACE, angiotensin-receptor blocker (ARB) or mineralocorticoid receptor antagonist (MRA) was not permitted). The primary composite outcome was end-stage kidney disease (ESKD), doubling of serum-creatinine level from baseline, or death from renal or CV cause. Pre-specified secondary outcomes (in hierarchical testing order) encompassed (a) a composite of CV death or hospitalization for HF (HHF), (b) a composite of CV death, MI, or stroke, (c) HHF, (d) a composite of ESKD, doubling of serum-creatinine level, or renal death, (e) CV death, (f) all-cause mortality, and (g) a composite of CV death, MI, stroke, HHF or hospitalization for unstable angina.

Administration of canagliflozin resulted in a 30% lower relative risk (HR 0.70 [95% CI 0.59–0.82]; p < 0.00001) of the primary composite outcome, compared to placebo. Effects were consistent for other renal outcomes (approximately 28–34% risk reduction) like ESKD, doubling of serum-creatinine, or renal death as well as across individual renal components of composite outcomes, including the doubling of serum-creatinine level and the exploratory outcome (dialysis, kidney transplantation, or renal death) [21]. Similarly, CV outcomes such as CV death, the composite of CV death or HHF, the composite of CV death, MI, or stroke, and the secondary outcome HHF were reduced by 20% to approximately 40% [21].

In terms of adverse events of special interest, there was no significant increase in lower limb amputation rate in the canagliflozin group compared to the control group (HR 1.11 [95% CI 0.79–1.56]). Previously published data of the CANVAS-Program had shown a small increase in atraumatic lower extremity amputations [30]. Rates of diabetic ketoacidosis were overall low (11 events for 2200 patients in the canagliflozin group, 1 event for 2197 patients in the control group), yet higher compared to placebo (HR 10.80 [95% CI 1.39–83.65]) [21].

The DAPA-HF study [22] investigated effects of dapagliflozin (10 mg daily) in 4744 patients with HFrEF over a median follow-up time of 1.52 years. Included were patients with diagnosed DM (41.8%) and without diagnosed DM (58.2%). Inclusion criteria encompassed an ejection fraction of ≤ 40% and New York Heart Association (NYHA) class II, III, or IV symptoms. In addition, participants were required to receive standard HF device-and drug therapy, participants with T2DM were able to continue their glucose-lowering medication, yet to be adjusted as required. Concomitant therapy with MRAs was encouraged. Primary outcome was a composite of worsening HF or death from CV causes. Key secondary outcomes included HHF or CV death [22].

Dapagliflozin showed significant improvement of the primary outcome (HR 0.74 [95% CI 0.65–0.85]; p < 0.001), with similar risk reductions detected for HHF (HR 0.70 [95% CI 0.59–0.83]) and CV death (HR 0.82 [95% CI 0.69–0.98]). Significant improvement of secondary outcomes was observed as well: risk for CV death or HHF (HR 0.75 [95% CI 0.65–0.85]; p < 0.001] and total number of HHF and CV deaths [HR 0.75 [95% CI 0.65–0.88]; p < 0.001) were significantly reduced. In addition, the increase in the Kansas City Cardiomyopathy Questionnaire Score (KCCQ; higher scores indicating fewer symptoms) was significant in the dapagliflozin group, compared to the placebo group (HR 1.18 [95% CI 1.11–1.26]; p < 0.001) [22]. Analysis of pre-specified subgroups revealed that patients with NYHA class II symptoms had the greatest risk reduction of the primary composite outcome as a result of dapagliflozin treatment. Furthermore, subgroup analysis demonstrated that patients with and without diagnosed T2DM had near to equal risk reductions (with T2DM HR 0.75 [95% CI 0.63–0.90]; without T2DM HR 0.73 [95% CI 0.60–0.88]). Similarly, no discrimination of risk reduction of the primary outcome could be observed according to the eGFR-value (< or ≥ 60 ml/min/1.73 m^2^) or MRA treatment at baseline [22].

No significant increase of adverse events of interest was observed for the dapagliflozin group compared to the placebo group. This encompassed comparable rates of volume depletion, renal adverse events, fractures, amputation, and major hypoglycaemia or diabetic ketoacidosis (the latter in patients with T2DM only) [22].

### GLP-1 receptor agonists

The REWIND trial [19] analysed CV effects of once-weekly administration of 1.5 mg dulaglutide in 9901 patients. Inclusion criteria comprised: (1) age ≥ 50 years with established T2DM (HbA1c ≤ 9.5% without lower limit, on stable doses of ≤ 2 oral glucose-lowering drugs with or without basal insulin if BMI ≥ 23 kg/m^2^) and with vascular disease; (2) age ≥ 55 years with established T2DM and vascular disease or subclinical vascular disease; (3) age ≥ 60 years with established T2DM and vascular disease or subclinical vascular disease or ≥ 2 CV risk factors [19]. The primary composite outcome was a composite of non-fatal MI, non-fatal stroke, and death from CV- or unknown causes. In addition, several secondary outcomes were analysed, comprising a composite clinical microvascular outcome which included diabetic retinopathy or renal disease.

Dulaglutide met its primary endpoint (HR 0.88 [95% CI 0.79–0.99); p = 0.026), thus significantly decreased 3P-MACE events over placebo. The pre-specified composite clinical microvascular outcome was significantly reduced in the active treatment group (HR 0.87 [95% CI 0.79–0.95]; p = 0.0020]) [19]. There were no significant differences in the subgroup analysis for the primary outcome with respect to age, sex, duration of diabetes, history of CVD, baseline HbA1c, and BMI when comparing dulaglutide to placebo [19]. An exploratory analysis of a composite renal outcome (new macroalbuminuria, sustained decline in eGFR of ≥ 30%, and chronic renal replacement therapy) demonstrated that dulaglutide significantly reduced the composite renal outcome (HR 0.85 [95% CI 0.77–0.93]), which remained significant if the sustained decline in eGFR was redefined to ≥ 40% and ≥ 50% [31].

The PIONEER-6 trial [20] investigated CV safety of oral semaglutide, the first FDA-approved oral GLP-1 RA [32]. PIONEER-6 included a total of 3183 patients randomized to receive oral semaglutide (14 mg daily after an 8 week run-in period of 3 mg and 7 mg oral semaglutide for 4 weeks, respectively) or placebo for a median follow-up period of 15.9 months. Eligible patients had an age ≥ 50 years and established CVD or CKD, or, if ≥ 60 years of age ≥ 1 CV risk factor. In total, 84.7% of patients were ≥ 50 years and had established CVD of CKD [20]. Primary outcome was a composite of death from CV causes (including undetermined causes of death), non-fatal MI, or non-fatal stroke. Secondary CV outcomes encompassed (a) an expanded composite outcome (primary endpoint plus unstable angina resulting in hospitalization or HF resulting in hospitalization), (b) a composite of death from any cause, non-fatal MI, or non-fatal stroke and (c) the individual components of the previously listed outcomes. Efficacy outcomes included HbA1c level, body weight, and lipid levels [20].

In patients receiving oral semaglutide the primary outcome was met, affirming non-inferiority of oral semaglutide (HR 0.79 [95% CI 0.57–1.11]; p < 0.001), yet not demonstrating superiority over placebo (p = 0.17) if added to standard of care [20]. Similarly, the expanded composite outcome (HR 0.82 [95% CI 0.61–1.10), the composite outcome made up of death from any cause, non-fatal MI, or non-fatal stroke (HR 0.77 [95% CI 0.56–1.05]), and the individual components of the latter two showed reductions in the oral semaglutide treatment arm (albeit not significant and to be considered exploratory due to non-significance of the superiority analysis of the primary outcome and hierarchical testing design) [20]. Exceptions were non-fatal MI and unstable angina resulting in hospitalization with a HR 1.18 [95% CI 0.73–1.90] and 1.56 [95% CI 0.60–4.01], respectively [20]. Analysis of efficacy outcomes demonstrated a reduction of HbA1c (− 0.7% difference between groups), body weight (− 3.4 kg difference between groups), and a modest decrease of LDL-cholesterol and triglycerides in favour of oral semaglutide over placebo [20].

### Angiotensin-receptor-neprilysin-inhibitors (ARNI)

In the previously published PARADIGM-HF [33] trial, a significant reduction of HFrEF was demonstrated for sacubitril-valsartan, compared to enalapril. The 2019 published PARAGON-HF trial [24] investigated the effects of sacubitril-valsartan compared to valsartan in patients with HFpEF. Eligible patients were 50 years or older, had signs and symptoms of HF (NYHA class II–IV), an ejection fraction of ≥ 45%, had evidence of structural heart disease, and diuretic therapy [24]. 43.5% of participants in the sacubitril-valsartan group had diabetes. The primary composite outcome was HHF and death from CV causes. Secondary outcomes encompassed change from baseline to 8 months in the clinical summary score on the KCCQ, change from baseline to 8 months in NYHA class, first occurrence of a decline in renal function, and death of any cause [24].

Sacubitril-valsartan did not meet the predetermined level of statistical significance (rate ratio (RR) 0.87 [95% CI 0.75–01.01]; p = 0.06), thus all other outcomes were considered exploratory. In general, positive effects of sacubitril-valsartan, compared to valsartan, were observed. These included a decreased rate of HHF (RR 0.85 [95% CI 0.72–1.00]), a higher percentage of patients with an improvement of 5 or more points in the KCCQ clinical summary score (odds ratio (OR) 1.30 [95% CI 1.04–1.61]), and a higher percentage of patients with an improvement of NYHA class (OR 1.45 [95% CI 1.13–1.86]). Renal composite outcome (death from renal failure, ESRD, or ≥ 50% eGFR decline) was reduced by 50% (HR 0.50 [95% CI 0.33–0.77]). No difference in death from any cause was observed (HR 0.97 [95% CI 0.84–1.13]). Subgroup analysis revealed a stronger effect of sacubitril-valsartan, compared to valsartan, in females vs. males, a left-ventricular ejection fraction of median ≤ 57% vs. > 57%, and MRA use vs. no MRA use, respectively. No differences were observed according to DM status [24].

Compared to valsartan, significant improvements of adverse events of special interest were observed: sacubitril-valsartan caused significantly less events of hypotension (systolic blood pressure < 100 mmHg), less events of elevated serum creatinine levels (≥ 2.0, ≥ 2.5, and ≥ 3.0 mg/dl, respectively), less events of elevated serum potassium levels (> 5.5 and > 6.0 mmol/l), and less angioedema events [24].

### Endothelin A receptor antagonists

The endothelin A receptor antagonist atrasentan has its history in the field of oncology, however, more recent clinical trials like the SONAR trial [23] have begun to investigate atrasentan in the context of renal disease. The SONAR trial differed from conventional outcome trials by using an enrichment design according to responsiveness to treatment (≥ 30% UACR reduction without substantial fluid retention) to investigate drug effects in the population with the highest expected benefit (responders), whilst aiming to minimize previously anticipated complications (risk of HF due to fluid retention). To assess effects in non-responders, a subgroup of non-responders (< 30% UACR without substantial fluid retention) was included [23]. Atrasentan significantly reduced the primary outcome (HR 0.65 [95% CI 0.49–0.88]; p = 0.0047), subdivided into a significant reduction of doubling of serum-creatinine (HR 0.61 [95% CI 0.43–0.87]; p = 0.0055) and a non-significant reduction of ESKD (HR 0.73 [95% CI 0.53–1.01]; p = 0.060) [23]. In non-responders, the primary renal outcome was not significantly reduced (HR 0.75 [95% CI 0.55–1.03]; p = 0.079). The authors concluded that using an enrichment strategy, designed to select patients most likely to benefit from a treatment may become a trendsetting option for future trials, in accordance with the concept of personalized medicine [23].

### Key topics discussed during the 5th CVOT Summit

#### SGLT-2i and GLP1-RA: a focus on heart and kidney

Including the CVOTs published in 2019, currently 7 CVOTs with GLP-1 RAs [13–17, 19, 20] and 5 CVOTs with SGLT-2is [10–12, 21, 22] have been published and their evidence analysed by multiple recent meta-analyses [34–38]. While it is clear that meta-analyses have to be considered with care, particularly with regard to varying inclusion criteria and pre-specified outcomes of the underlying studies, all recent meta-analyses convey the clear message that both GLP-1 RAs and SGLT-2is consistently show cardiovascular and renal benefits [34–38], yet with slightly different benefits, as displayed in currently recommended treatment algorithms and guidelines [39, 40].

A meta-analysis encompassing the trials EMPA-REG Outcome [12], CANVAS Program [10], and DECLARE-TIMI 58 [11] compared CV effects of SGLT-2is [36]. Overall, SGLT-2is in these trials reduced the risk of 3P-MACE significantly (HR 0.89 [95% CI 0.83–0.96]; p = 0.0014), with overall significant reduction of MI (HR 0.89 [95% CI 0.80–0.98]; p = 0.0177) and CV death (HR 0.84 [95% CI 0.75–0.94]; p = 0.0023), yet without a significant effect on stroke (HR 0.97 [95% CI 0.86–1.10]) [36]. In contrast to GLP-1 RAs, SGLT-2is markedly reduced the risk for the composite of CV death or HHF (HR 0.77 [95% CI 0.71–0.84]; p < 0.0001), and HHF (HR 0.69 [0.61–0.79]; p < 0.0001) [36]. In both meta-analyses, effects of treatment (GLP-1 RA or SGLT-2i) were driven by the patient groups with prior CVD, whereas in either case no significant risk reduction was observed for patients with multiple risk factors [34, 36].

A follow up meta-analyses by Neuen et al. [38] investigated the overall renal effects observed in the SGLT-2i outcome trials CREDENCE [21], DECLARE-TIMI 58 [11], the CANVAS Program [10], and EMPA-REG OUTCOME [12]. Even though a high number of patients was included in the latter three CVOTs, most participants were at low risk of clinically relevant kidney outcomes. With the publication and inclusion of CREDENCE, a trial primarily powered for renal outcomes in patients with established DKD [21], the number of patients with renal disease increased substantially, allowing for a better powered meta-analysis [38]. They showed significant overall risk reduction of dialysis, transplantation, or death due to kidney disease (HR 0.67 [95% CI 0.52–0.86]; p = 0.0019), ESKD (HR 0.65 [95% CI 0.53–0.81]; p < 0.0001), and substantial loss of kidney function, ESKD, or death due to kidney disease (HR 0.58 [95% CI 0.51–0.66]; p < 0.0001) [38]. Similarly, an overall significantly reduced risk of acute kidney injury (HR 0.75 [95% CI 0.66–0.85]; p < 0.0001) was demonstrated. A stratification of the combined outcome of substantial loss of kidney function, ESKD, or death due to kidney disease by baseline eGFR, UACR and use of RAS blockade demonstrated that overall, significant risk reduction was observed to be independent from eGFR (eGFR of < 45, 45– < 60, 60– < 90, or ≥ 90 ml/min/1.73 m^2^) and UACR (UACR of > 30, 30–300, or > 300 mg/g), yet with comparably better risk reduction upon concomitant RAS blockade [38].

One of the most recent meta-analyses, by Kristensen et al. [34], including all 7 GLP-1 RA CVOTs, demonstrated that treatment with GLP-1 RA resulted in a significant overall risk reduction of the CV endpoints of 3P-MACE (HR 0.88 [95% CI 0.82–0.94]; p < 0.0001), and its components CV death (HR 0.88 [95% CI 0.81–0.96]; p = 0.003), fatal or non-fatal MI (HR 0.91 [95% CI 0.84–1.00]; p = 0.043), and fatal or non-fatal stroke (HR 0.84 [95% CI 0.76–0.93]; p < 0.0001) [34]. For the first time, an overall risk reduction for HHF (HR 0.91 [95% CI 0.83–0.99]; p = 0.0028) was shown. Overall, also all-cause mortality (HR 0.88 [95% CI 0.83–0.95]; p = 0.001) was reduced significantly. In terms of renal risk reduction, the risk for the renal outcome of composite kidney outcome including macroalbuminuria (HR 0.83 [95% CI 0.78–0.89]; p < 0.0001) was significantly reduced. Liraglutide, semaglutide, and dulaglutide and their respective studies seem to be the three major driving agents within the overall composite renal outcome including macroalbuminuria in the corresponding meta-analysis [34]. When looked at in more detail in respective trials, the composite renal outcome was to a large extent driven by a reduction of the risk of new onset of persistent macroalbuminuria [15, 17, 19].

Results and observations from CVOTs have strongly impacted relevant guidelines—already in 2018, the ADA/EASD consensus recommendation incorporated latest data from CVOTs and redefined the treatment algorithm with metformin as first line pharmaceutical therapy, followed by second line pharmaceutical treatment according to present comorbidities such as ASCVD, HF, or CKD [40]. Recently, the updated 2019 ESC Guidelines on diabetes, pre-diabetes, and cardiovascular diseases developed in collaboration with the EASD went a step further and incorporated GLP-1 RAs and/or SGLT-2is as first line pharmaceutical therapy in drug-naïve patients with ASCVD, or high/very high CV risk (target organ damage or multiple risk factors) [39].

### Primary care in diabetes management

It was acknowledged that GLP-1 RAs and SGLT-2is become more relevant to a broader population, also including increasing application in primary care. While most CVOTs have included a large population of patients at high CV or renal risk which may more frequently present to a specialist physician rather than a primary care physician, two of the published CVOTs have included large primary prevention cohorts: DECLARE-TIMI 58 [11] and REWIND [19]. DECLARE-TIMI 58 investigated the effect of dapagliflozin in a trial population of which 59.5% (10,186 patients) had no established CVD and demonstrated a 16% risk reduction of 3P-MACE (non-significant) in the multiple risk factors population [11]. In terms of primary prevention of kidney disease, results of SGLT-2 inhibitor CVOTs are backed up by the real-world data of CVD-REAL 3 [41]. The study investigated renal and CV outcomes of SGLT-2is in a total of 71,122 treatment initiation episodes from 65,231 individual subjects in 5 different countries (Israel, U.K., Italy, Taiwan, and Japan), compared to other glucose-lowering drugs. The majority of treatment initiation episodes (ca. 52%) were in patients with preserved kidney function (eGFR > 90 ml/min/1.73 m^2^) and ca. 38% of treatment initiation episodes in patients with an eGFR of 60 to < 90 ml/min/1.73 m^2^, thus making the study relevant to primary care [41]. Overall, CVD-REAL 3 demonstrated significantly less renal events in the SGLT-2i treatment group across the spectrum of assessed renal outcomes (e.g. composite of 50% eGFR decline or ESKD (HR 0.49 [95% CI 0.35–0.67]; p < 0.0001), or ESKD alone (HR 0.33 [95% CI 0.16–0.68]; p = 0.0024), with consistent results across pre-specified subgroups, like concomitant use of ACEis or ARBs. HHF (HR 0.60 [95% CI 0.47–0.76]; p < 0.0001) and all-cause mortality (HR 0.55 [95% CI 0.48–0.64]; p < 0.0001) were decreased compared to other glucose-lowering drugs [41].

REWIND investigated the effect of dulaglutide in a population in which 68.5% (6793 patients) had not established CVD [19], and reported a 13% risk reduction of 3P-MACE (non-significant) in the multiple risk factors population [11, 19]. A recent exploratory analysis [42] of REWIND reported a significantly reduced risk of non-fatal stroke (HR 0.76 [95% CI 0.61–0.95]; p = 0.017) and ischaemic stroke (HR 0.75 [95% CI 0.59–0.94); p = 0.0115), and no significant effects on fatal stroke, haemorrhagic stroke, or stroke of unknown type when comparing dulaglutide to placebo [42]. When analysing this in context of primary prevention, a significant effect was only observed for participants with previous CVD, however, a non-significant risk reduction of 20% (HR 0.80 [95% CI 0.61–1.06]) was observed in primary prevention [42]. Even though more studies are needed on the potential benefits of GLP-1 RAs and SGLT-2is in a primary prevention population [43, 44], this is a first step towards making CVOTs, their outcomes and medications tested relevant for both broad primary and secondary prevention populations, and thus both, specialist and primary care physicians.

## Conclusion

The 5th CVOT Summit discussed key results of recently completed and published CVOTs in T2DM (CAROLINA, PIONEER-6, and REWIND) as well as two trials designed to evaluate specifically renal outcomes (CREDENCE) and HF outcomes (DAPA-HF) in an interactive, multi-disciplinary format. The summit considered latest data on possible mechanistic backgrounds, as well as potential and limitations of the recently published CVOTs and their implications in current guidelines for specialist and primary care provided to individuals with DM. In-depth discussions and presentations of upcoming CVOTs, renal and HF trials like DAPA-CKD, EMPA-Kidney, VERTIS-CV, Emperor-Reduced, and Emperor-Preserved will be resumed at the 6th CVOT Summit, which will be held in Munich from 29 to 30 October 2020 (https://www.cvot.org).

## Data Availability

Data sharing not applicable to this article as no datasets were generated during the current study.

## Abbreviations

3P(4P)-MACE: 3-point (4-point) major adverse cardiovascular event
ACE: Angiotensin-converting-enzyme
ARB: Angiotensin-receptor blocker
ASCVD: Atherosclerotic cardiovascular disease
CHD: Coronary heart disease
CI: Confidence interval
CKD: Chronic kidney disease
CVD: Cardiovascular disease
CV: Cardiovascular
CVOT: Cardiovascular outcome trial
DCVD: Diabetes and Cardiovascular Disease EASD Study Group
EDNSG: European Diabetic Nephropathy Study Group
DKD: Diabetic kidney disease
DM: Diabetes mellitus
DPP-4i: Dipeptidyl-peptidase 4 inhibitor
EASD: European Association for the Study of Diabetes
ESKD: End-stage kidney disease
FDA: U.S. Food and Drug Administration
(e)GFR: (estimated) Glomerular filtration rate
HF: Heart failure
HFpEF: Heart failure with preserved ejection fraction
HFrEF: Heart failure with reserved ejection fraction
HHF: Hospitalization for heart failure
HR: Hazard ratio
IDF: International diabetes federation
GLP-1 RA: Glucagon-like peptide 1 receptor agonist
KCCQ: Kansas City Cardiomyopathy Questionnaire
LDL: Low-density lipoprotein
MI: Myocardial infarction
MRA: Mineralocorticoid receptor antagonist
NYHA: New York Heart Association
OR: Odds ratio
PCDE: Primary Care Diabetes Europe
RR: Rate ratio
SGLT-2i: Sodium/glucose cotransporter 2 inhibitor
T2DM: Type 2 diabetes mellitus
UACR: Urine albumin-creatinine ratio
